# Generative adversarial local density-based unsupervised anomaly detection

**DOI:** 10.1371/journal.pone.0315721

**Published:** 2025-01-24

**Authors:** Xinliang Li, Jianmin Peng, Wenjing Li, Zhiping Song, Xusheng Du

**Affiliations:** 1 Chongqing College of International Business and Economics, ChongQing, China; 2 People’s Hospital of Xinjiang Uygur Autonomous Region, Urumqi, China; 3 School of Information Science and Engineering, Xinjiang University, Urumqi, China; Prince Sultan University, SAUDI ARABIA

## Abstract

Anomaly detection is crucial in areas such as financial fraud identification, cybersecurity defense, and health monitoring, as it directly affects the accuracy and security of decision-making. Existing generative adversarial nets (GANs)-based anomaly detection methods overlook the importance of local density, limiting their effectiveness in detecting anomaly objects in complex data distributions. To address this challenge, we introduce a generative adversarial local density-based anomaly detection (GALD) method, which combines the data distribution modeling capabilities of GANs with local synthetic density analysis. This approach not only considers different data distributions but also incorporates neighborhood relationships, enhancing anomaly detection accuracy. First, by utilizing the adversarial process of GANs, including the loss function and the rarity of anomaly objects, we constrain the generator to primarily fit the probability distribution of normal objects during the unsupervised training process; Subsequently, a synthetic dataset is sampled from the generator, and the local synthetic density, which is defined by measuring the inverse of the sum of distances between a data point and all objects in its synthetic neighborhood, is calculated; Finally, the objects that show substantial density deviations from the synthetic data are classified as anomaly objects. Extensive experiments on seven real-world datasets from various domains, including medical diagnostics, industrial monitoring, and material analysis, were conducted using seven state-of-the-art anomaly detection methods as benchmarks. The GALD method achieved an average AUC of 0.874 and an accuracy of 94.34%, outperforming the second-best method by 7.2% and 6%, respectively.

## 1. Introduction

Anomaly detection is a critically important task in the field of data mining, widely applied in various domains such as financial fraud detection [1], cybersecurity [2], healthcare [3], and industrial monitoring [4]. The essence of anomaly detection tasks lies in accurately identifying data objects that deviate from normal behavior patterns [5]. These anomaly objects often represent crucial indicators of significant events or potential issues within systems. Therefore, the accuracy of anomaly detection is not only crucial for enhancing operational efficiency across various domains but also serves as a cornerstone for ensuring effective decision-making processes and system security [6].

As data collection capabilities continue to strengthen, methods for anomaly detection are also advancing. In the early stages, anomaly detection primarily relied on simple statistical analysis techniques to identify anomalies, such as setting thresholds or conducting basic statistical tests [7]. However, these methods often assume that data follow a specific distribution, such as a normal distribution. With the increasing complexity and diversity of data, traditional methods are gradually showing limitations [8]. The introduction of machine learning algorithms has significantly expanded the application scope of anomaly detection while enhancing detection efficiency and accuracy. For instance, support vector machines (SVM) [9], decision trees [10], and other models can effectively handle and analyze complex datasets, freeing anomaly detection from strict statistical assumptions and manual feature extraction limitations. Despite the significant advantages of machine learning methods over traditional approaches, their performance heavily relies on the quality and quantity of training data. Moreover, they may not be responsive to newly emerging anomaly types, limiting their generalization ability [11].

In recent years, the application of deep learning methods in anomaly detection has been increasingly prominent. Their primary advantage lies in their ability to handle highly complex and nonlinear data patterns, thereby enhancing the detection and generalization capabilities for anomalies [12]. However, existing deep learning methods often struggle to detect anomaly objects with complex distributions that closely resemble normal object distributions [13]. The introduction of generative adversarial networks has brought a new research paradigm to anomaly detection. GANs leverage an adversarial process between a generator and a discriminator to learn the latent distribution of data, offering significant advantages in handling complex data distributions [14]. The generator aims to create synthetic data that mimics the real data distribution, while the discriminator attempts to distinguish between real and synthetic data. This adversarial setup forces the generator to learn intricate, nonlinear features of the data, enabling GANs to approximate highly complex and non-Gaussian distributions. The continuous feedback loop between the generator and discriminator allows GANs to progressively improve their modeling of complex patterns that traditional models often struggle to capture.

However, while GANs are adept at learning and generating synthetic data that closely follows the latent normal data distribution, distinguishing between low deviation anomaly objects and normal ones can still be challenging. This is where the calculation of local synthetic density plays a crucial role. By calculating the local synthetic density between objects in the original data and their synthetic neighbors, we can provide a more refined measure of how well each data point aligns with the synthetic data distribution generated by the GANs. Unlike simple neighborhood relationships, which often fail to differentiate anomaly objects in complex high-dimensional spaces due to their reliance on fixed distance metrics, local density synthetic offers a more nuanced analysis of how isolated or well-integrated a data point is within its local region. This approach effectively enhances the ability of GANs-based methods to identify anomalies, as it captures subtle deviations that simple neighborhood-based methods often overlook. Hence, the integration of local synthetic density with GANs allows for a more robust identification of anomalies that are less apparent under traditional neighborhood approaches. Therefore, we propose a GANs with local synthetic density-based anomaly detection method. The main contributions are summarized as follows:

(1): The proposed GALD method effectively leverages the distribution fitting capabilities of GANs, allowing the generator to learn the underlying normal data distribution, while also incorporating local density measures to better identify anomaly objects. This dual approach ensures that even subtle deviations are effectively detected, particularly when anomaly objects are hidden within complex distribution patterns.

(2): The GALD method calculates the local synthetic density by comparing the density differences between the generated synthetic data and the original data. This local density calculation provides a more precision view of the data distribution, allowing the model to capture subtle changes that indicate the presence of anomalies, thus significantly enhancing detection accuracy in high-dimensional and imbalanced datasets.

(3): In class-imbalanced anomaly detection tasks, GANs tend to prioritize fitting the majority class (normal objects) to minimize the loss under adversarial training. The GALD method by using the synthetic data generated by GANs as fake-normal objects and incorporating local synthetic density to better detect minority anomaly objects. This approach ensures a more robust and precise anomaly detection process, effectively overcoming the challenges of class imbalance.

## 2. Related work

This section provides an overview of existing anomaly detection methods, including classical statistical and machine learning approaches, as well as the application of deep learning and generative adversarial networks in anomaly detection. By comparing the strengths and weaknesses of these methods, it establishes a theoretical foundation and background support for subsequent research.

### 2.1 Classical anomaly detection methods

Classic anomaly detection methods can be broadly categorized into statistical-based, clustering-based, density-based [15], and distance-based [16] detection methods. Statistical-based methods identify anomaly objects by calculating statistical metrics such as mean and standard deviation. However, these methods assume data follow a specific distribution and are sensitive to extreme values, making them less effective for handling multivariate data. Cluster-based methods detect anomaly objects by partitioning data points into different clusters. For instance, the K-Means clustering method divides data into clusters, where data points not belonging to any major cluster or belonging to small and sparse clusters are more likely considered anomaly objects [17]. However, these methods are sensitive to parameters, perform poorly on high-dimensional data, and struggle with handling clusters of complex shapes [18]. Density-based methods identify anomaly objects by estimating the density of data points in their vicinity. The typical Local Outlier Factor (LOF) method calculates the local density for each data point, where points in low-density regions are considered potential anomaly objects or abnormal activities [19]. However, density-based methods are computationally intensive, challenging in parameter selection, and sensitive to points at the boundaries of the data distribution. Distance-based methods identify anomaly objects by calculating distances between data points. The typical K-Nearest Neighbors (KNN) method, for example, identifies anomaly objects by computing the distance between a target data point and its nearest neighbors, marking data points with unusually large distances as anomaly objects [20]. While straightforward and intuitive, these methods often perform poorly with high-dimensional data. Table 1 summarizes these classical methods.

**Table 1:** Classical anomaly detection methods. The advantages and disadvantages of classical anomaly detection methods such as statistics, clustering, density and distance are summarized to help understand their applicability.

Algorithm name	Advantage	Disadvantage
Statistical-based	Simple and effective for data following a known distribution.	Assumes a specific distribution; sensitive to extreme values; less effective for multivariate data.
Cluster-based (K-Means)	Partitions data into clusters, identifies anomalies as points in sparse clusters.	Sensitive to parameter settings; performs poorly on high-dimensional data; struggles with complex shapes.
Density-based (LOF)	Estimates local density to identify anomalies; effective for finding outliers in low-density regions.	Computationally intensive; challenging parameter selection; sensitive to boundary points.
Distance-based (KNN)	Straightforward and intuitive; effective for low-dimensional data.	Poor performance in high-dimensional data; computationally expensive for large datasets.

### 2.2 Deep learning-based anomaly detection method

As data volumes and computational power expand, deep learning algorithms have become increasingly effective for handling high-dimensional data and complex pattern recognition. These methods build complex neural network models that autonomously extract high-dimensional features from data, facilitating anomaly detection. Key techniques in deep learning-based anomaly detection include Autoencoders [21], Convolutional Neural Networks (CNN) [22], Long Short-Term Memory networks (LSTM) [23], and Graph Neural Networks (GNN) [24].

Methods based on autoencoders leverage the ability of autoencoders to reconstruct data, demonstrating significant potential in industrial anomaly detection. However, their performance depends on the quality and quantity of the data. When the proportion of anomaly data increases or the data quality is low, the effectiveness of anomaly detection based on autoencoders tends to decrease. Additionally, anomaly detection methods based on autoencoders are particularly sensitive to the choice of threshold for reconstruction errors [25]. Convolutional Neural Networks (CNN) have been utilized in recent years for anomaly detection in time series and multidimensional data, owing to their ability to extract local features from data and identify complex patterns and anomaly objects through their multi-layered structure. However, CNN models require complex preprocessing steps to ensure data quality, which can lead to increased computational costs and training times when handling large datasets [26]. Recurrent Neural Networks (RNN) and Long Short-Term Memory networks (LSTM) are suitable for learning from long sequential data and can identify anomaly objects when data behaviors deviate from normal patterns [27]. However, the performance of these models largely depends on the setting of hyperparameters, and the network training can be unstable when dealing with long sequences of data [28]. Graph Neural Networks (GNNs) achieve effective results in anomaly detection tasks by learning the relationships between nodes and edges in graph-structured data, coupled with message-passing techniques in hidden layers. However, the training process for GNN is complex, and constructing graphs for large-scale data incurs high computational and training costs [29]. Additionally, the model’s sensitivity to changes in graph structure can impact the stability of detection results. Table 2 summarizes these deep learning-based methods.

**Table 2:** Deep learning-based anomaly detection method. The capabilities and limitations of deep learning methods in dealing with complex data distributions are compared.

Algorithm name	Advantage	Disadvantage
Autoencoder	Learns feature representations and reconstructs data; effective for unsupervised learning.	Sensitive to the quality of data; effectiveness drops with high anomaly ratios; threshold choice impacts performance.
CNN	Extracts local features and identifies complex patterns; effective for time series and multidimensional data.	Requires complex preprocessing; high computational cost and long training times for large datasets.
RNN/LSTM	Effective for sequential data; learns from deviations in time-series patterns.	Sensitive to hyperparameters; unstable training for long sequences; computationally intensive.
GNN	Learns relationships in graph-structured data; uses message-passing for anomaly detection.	High computational cost for large graphs; sensitive to graph structure changes; complex training process.

### 2.3 GANs-based anomaly detection method

The anomaly detection method based on generative adversarial networks has received widespread attention in recent years. This approach identifies anomaly data through the adversarial training of a generator and a discriminator. The generator generator objects similar to the training data, while the discriminator evaluates the authenticity of these objects. When the input objects cannot be reconstructed by the generator or is recognized as an anomaly objects by the discriminator, it is considered an anomaly [30]. In this way, GANs models not only generate new data objects to augment the training set but also help the model better understand the normal and anomaly patterns within the data.

The TGAN(Transformer-with-GANs) generates synthetic data similar to real tabular data using GANs, and then uses reconstruction error to detect anomaly objects, achieving high detection accuracy [31]. However, existing GANs-based anomaly detection methods also have several drawbacks. For instance, the anomaly generative adversarial network (AnoGAN) train GANs with unlabeled data and detects outliers by comparing the differences between real and generated data [32]. However, its training process is often unstable and has limitations, especially when dealing with high-dimensional data. f-AnoGAN(fast-AnoGAN) improves the efficiency and accuracy of AnoGAN by introducing a fast-training encoder network that maps data to the latent space of GANs. Although it enhances the efficiency of anomaly detection, f-AnoGAN still does not address the inherent training instability of GANs [33]. GANomaly employs an encoder-decoder-encoder structure to generate latent space representations of data and identifies outliers by comparing the differences between the original input and the reconstruction. However, its effectiveness can be limited for complex data structures, especially when there is an insufficient number of normal objects [34]. Multiple(Single)-Objective Generative Adversarial Active Learning (MO-GAAL, SO-GAAL) is an innovative anomaly detection method that introduces a multi-generator structure and an active learning strategy based on GANs. It leverages multiple generators working collaboratively to generate richly informative potential anomaly objects, significantly enhancing anomaly detection performance and robustness on complex datasets. Although MO-GAAL uses a multi-generator structure to avoid mode collapse of a single generator, it still faces issues such as insufficient coordination among generators or imbalanced generation distributions. Additionally, its performance and effectiveness may be highly influenced by the choice of hyperparameters [35]. STEP-GAN (step-by-step training method for multi generator GANs) is an improved generative adversarial network approach that leverages multiple generators interacting step-by-step with a discriminator to learn different modes of the distribution of task-specific normal data, thereby simulating potential anomaly distributions and mitigating the mode collapse problem. Its limitation lies in the fact that, since the model is entirely trained on normal data, its ability to detect unseen or significantly different anomaly patterns may be limited [36]. Table 3 summarizes the methods based on GANs.

**Table 3:** Generative adversarial nets-based anomaly detection. The features and challenges of different GANs-based anomaly detection methods are listed, highlighting their respective detection effectiveness and shortcomings.

Algorithm name	Advantage	Disadvantage
TGAN	Generates synthetic data like real tabular data; Uses reconstruction error for high anomaly detection accuracy.	Difficult handle highly complex or high-dimensional data effectively
AnoGAN	Uses GANs trained with unlabeled data to detect anomalies by comparing real and generated data.	Training process is often unstable, especially in high-dimensional settings.
f-AnoGAN	Improves efficiency and accuracy by introducing a fast-training encoder.	Does not fully address GAN’s inherent training instability.
GANomaly	Employs an encoder-decoder-encoder structure for latent space representation and anomaly detection.	Limited effectiveness for complex data structures, particularly when normal samples are insufficient.
MO-GAAL	Uses multiple generators to collaboratively generate potential anomaly objects, improving robustness.	Issues with insufficient generator coordination and hyperparameter sensitivity.
SO-GAAL	Introduces an active learning strategy based on GANs to enhance anomaly detection.	Suffers from imbalanced generation distributions and is sensitive to hyperparameter choices.
STEP-GAN	Learns modified versions of task-specific normal data using multiple generators to simulate possible anomalies, reducing mode collapse.	Limited by reliance on normal data, potentially affecting detection of unseen anomaly types.

## 3. Methodology

**Fig 1:**
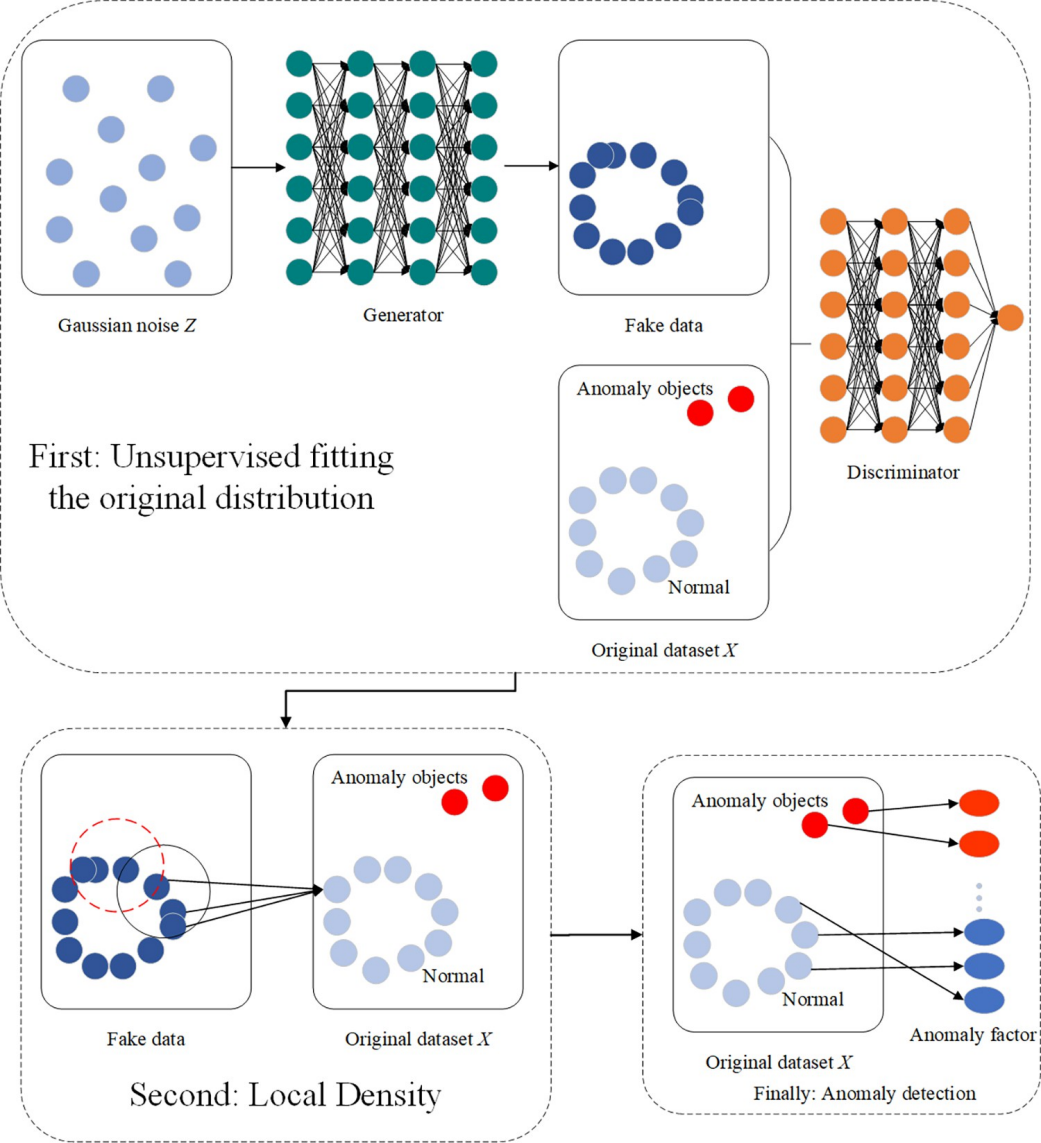
The entire structure of GALD. The overall flow of the GALD method is shown, including the interaction of the generator and the discriminator, and how anomaly detection is achieved by local synthesis of the density.

Confronted with complex data distributions, existing anomaly detection methods often struggle due to their reliance on statistical assumptions and neighborhood relations. This limitation makes it difficult to effectively fit the patterns of complex distributions. To address this challenge, we have proposed the GALD method. As illustrated in Fig 1, GALD constrains its generator with a loss function to better fit the probability distribution of more frequent objects in the original dataset, then compares the neighborhood density similarity between the original data and the synthetic data produced by the generator, with greater deviations indicating a higher likelihood of anomaly objects.

### 3.1 Unsupervised learning data distribution

In anomaly detection tasks, datasets typically exhibit two distinct distribution patterns: the majority being normal objects distributions and the minority being anomaly objects distributions. While the GAN network is a special type of neural network that includes a generator module and a discriminator module. The primary function of the generator is to receive random vectors (typically from a Gaussian distribution) and generate synthetic data objects consistent with the original data distribution.

When the GAN processes input data containing both normal and anomaly objects, its generator, constrained by the loss function, prioritizes learning the latent distribution of the overwhelmingly predominant normal objects in the original data, thus minimizing errors to the greatest extent. The discriminator, on the other hand, is responsible for distinguishing which of the input data comes from the original data and which comes from the synthesized data of the generator. The model continuously updates parameters during training, with the generator and discriminator being alternately optimized. The training objective of the GAN can be expressed by the following Eq (1).:

$$\underset{G}{min}\underset{D}{max}V(G,D)=E_{x}∼p_{data}(x)[\log\left(D(x)\right)]+E_{z}∼p_{\left(z\right)}[\log\left(1-D(G(z))\right)]$$ (1)

**Fig 2:**
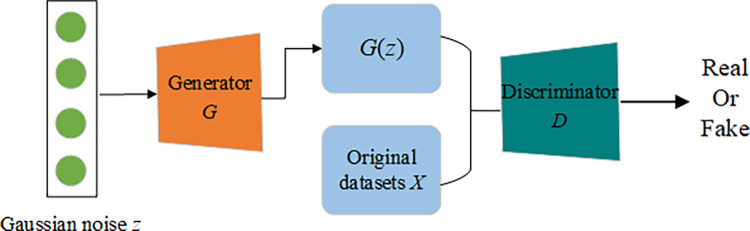
The training framework of GANs. Describes how the GANs model learns the distribution of original data through adversarial training between a generator and a discriminator.

In Eq (1), *D*(*x*) denotes the output of the discriminator *D* for a given object *x* of original data. If *x* from the original dataset, the discriminator should output a value close to 1, indicating that it considers the object to be “real”. *G*(*z*) denotes the data object synthesized by the generator. *D*(*G*(*z*)) is the probability assigned by the discriminator to classify the generated sample as real, with an expected value of 0. Fig 2 represents the training framework of the GANs.

The respective training objectives of the discriminator and generator are shown in Eqs 2 and 3, *x*_*i*_ denote the *i*-th object within the original data containing *m* objects, *k* represents the amount of generated objects, and *z*_*j*_ represents the *j*-th generated object.


$$maxV_{D}=\sum_{i=1}^{m}log(D(x_{i}))+\sum_{j=1}^{k}log(1-D(G(z_{j})))$$ (2)

$$minV_{G}=\sum_{j=1}^{k}log(1-D(G(z_{j})))$$ (3)

**Fig 3:**
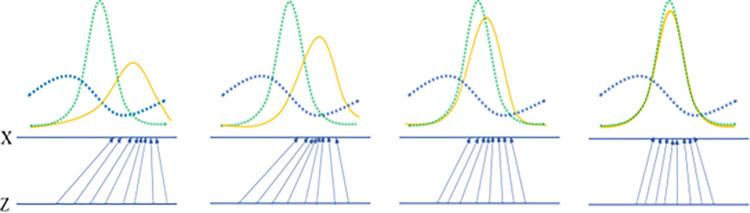
Generator fitting the distribution of the original dataset *X*. Demonstrates the generator’s ability to fit the original data distribution by progressively generating samples that more closely match it during adversarial training.

The generator optimizes its output by minimizing the objective function in Eq 3. During this optimization process, due to the dominance of normal objects, the generator predominantly learns how to generate samples like normal objects. Since anomaly objects are relatively rare, they have limited impact on the loss function, which leads the generator to focus primarily on the distribution characteristics of normal objects. Let the original datasets be *X* = {*x*_1_, *x*_2_, *x*_3_,…, *x*_*n*_}∈*R*^d*n^, where *x*_*i*_ represents any object in *X*. Fig 3 illustrates the generator fitting the original data distribution during adversarial training of the GANs.

During the training process of GANs, the generator optimizes the generated data by minimizing the discriminator’s judgment error. Since normal objects are the majority in the dataset, the generator is primarily influenced by these normal samples during optimization, naturally tending to learn and fit the distribution of normal data. At the same time, the rarity of anomaly objects means they have a relatively small impact on the loss function, causing the generator to focus more on generating data like normal samples. Additionally, in the early stages of training, the discriminator can easily distinguish between normal and anomaly data, which forces the generator to gradually produce samples that are closer to normal data in order to fool the discriminator, thus prioritizing the learning of normal objects distribution.

In Fig 3, the blue curve represents the distribution of anomaly objects, the yellow curve represents the distribution of synthetic data by the generator, and the green curve represents the distribution of normal objects. In Fig 3, the lower part of each subplot represents the noise area *Z*, and the arrows indicate the mapping relationship between the data input to the generator and the original data *X* distribution. Fig 3A shows the noise distribution mapped by the generator at random initialization, while Fig 3B–3D show the noise distribution mapped by the generator *G* after continuous parameter updates.

**Fig 4:**
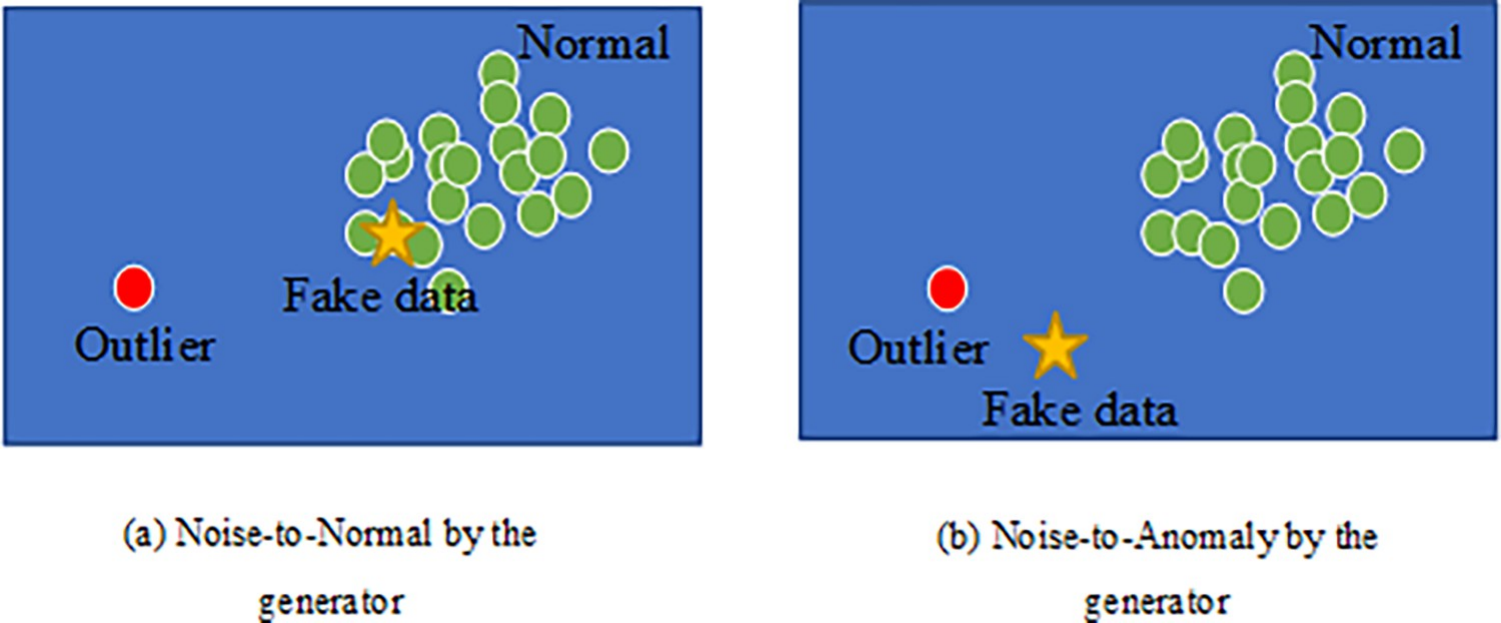
Generator maps noise to two potential latent distributions. It is illustrated how the generator converts input noise into normal or anomaly objects in the latent distribution for effective anomaly detection.

Since normal and anomaly objects in the original dataset are samples from two different latent distributions, the generator has two distinct mapping methods for the input noise to minimize the error in Eq 3, as shown in Fig 4.

As shown in Fig 4(A), when the objects generated by the generator closely resemble normal objects, it becomes increasingly difficult for the discriminator to distinguish between the synthetic and the original data. The discriminator struggles to differentiate between them using a simple boundary. On the contrary, Fig 4(B) depicts objects generated by the generator that align with the distribution of anomaly objects. In such a case, the discriminator identifies it easier to differentiate between fake and original data. Obviously, this outcome does not align with the training objectives of the generator. Thus, in unsupervised learning of the original data distribution, the GAN network primarily focuses on capturing the distribution of normal objects.

**Method 1** Unsupervised fitting the data distribution

**Input:** Original dataset ***X***, Learning rate***γ***, Number of iterations ***n***, sample size ***m***.

**Output:** Fake data

1. **for** *n* = 1:*m* **do**

2. **for** *k* = 1 **do**

3.  Sample *m* objects from *X*.

4.  Sample *m* noise objects {*z1*, *z2*, *…*, *zi*} from *Pz*(*z*).

5.  #Update discriminator by increasing random gradient:

6.  $\nabla_{\theta d}\frac{1}{m}\Sigma_{i=1}^{m}[logD(x_{i})+log(1-D(G(z_{i})))]$

7. **end for**

8. Sample *m* noise samples {*z1*, *z2*, *…*, *zi*} from *Pz*(*z*).

9. #Update generator by increasing random gradient:

10.  $\nabla_{\theta g}\frac{1}{m}\sum_{i=1}^{m}\;log(1-D(G(z_{i})))$

11. **end for**

12. **return** Fake data

### 3.2 Local synthetic density

Local synthetic density refers to the similarity in regional density between the original data and the data synthesized by the generator. In unsupervised training, the generator synthetic the representations of normal objects found in the original data. The introduction of local synthetic density aims to address the limitations of GANs in fitting highly imbalanced data distributions. By calculating the local synthetic density, we aim to provide a more refined measure of how well each data point aligns with the synthetic data distribution generated by the GANs. This approach helps to enhance anomaly detection by capturing subtle deviations that traditional GANs-based methods may overlook, particularly in complex and imbalanced data settings.

If an original data object and its synthetic neighbor objects exhibit high similarity in terms of local density, it indicates that the object conforms well to the distribution of normal objects. Conversely, if the local density is significantly different, the object is more likely to be an anomaly object.

Definition 1: Neighborhood

Let *FD* represent the set of synthetic data through the GANs, define the neighborhood of *Xi*, denoted as *Nk*(*Xi*), as the collection of the closest neighbors in *FD* to the data point *Xi*.


Nk(Xi)={FDi∈FD|dist(FDi,Xi)≤k−distance(Xi)} (4)

*FDi* represents any object in the synthetic data, *dist*(*FDi*,*Xi*) denotes the Euclidean distance between *FDi* and *Xi*, and *k*-distance(*Xi*) represents the distance between *Xi* and the *k*-th nearest object in the synthetic data. The calculation method for *dist*(*FDi*,*Xi*) is given by Eq 5:

$$dist(X_{i},FD_{i})=\sqrt{(X_{i1}-FD_{i1}{)}^{2}+(X_{i2}-FD_{i2}{)}^{2}+,\ldots ,+(X_{id}-FD_{id}{)}^{2}}$$ (5)

Definition 2: Local synthetic density (LSD)

Local synthetic density is particularly useful in anomaly detection as anomaly objects often exist in sparse regions where the local synthetic density is low. The local synthetic density of an object *X*_*i*_ in the original data *X* is calculated as the reciprocal of the sum of distances between *X*_*i*_ and the objects in its synthetic neighborhood *N*_*k*_ (*X*_*i*_). A higher local synthetic density suggests that the object is situated near the central region of the synthetic data, while a lower density indicates that the object is positioned further from the center. The formula for calculating local synthetic density is as follows Eq 6:

$$LSD(X_{i})=\frac{1}{\sum_{FD_{j}\in N_{k}(X_{i})}dist(X_{i},FD_{j})}$$ (6)

**Fig 5:**
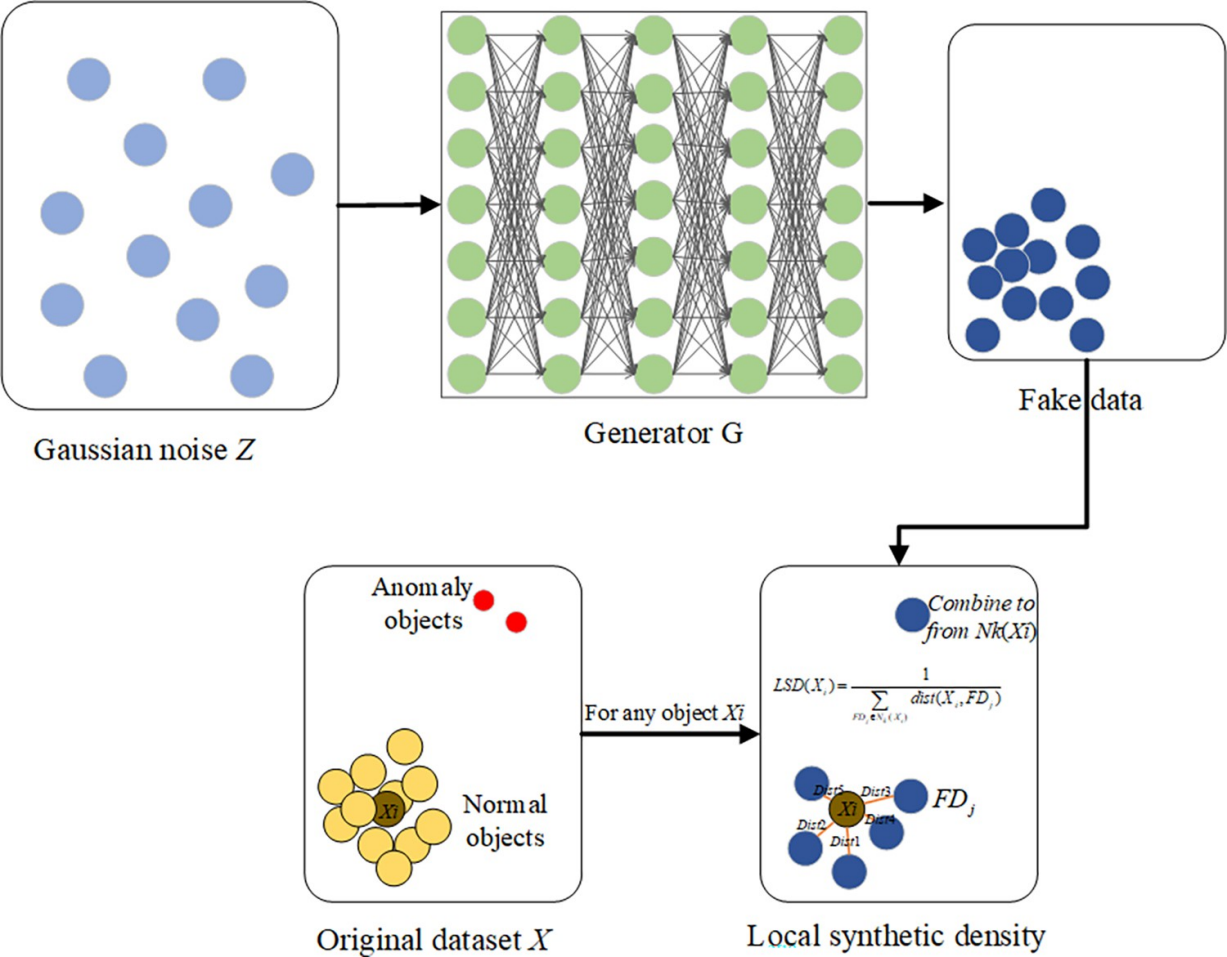
Local synthetic density. The process of how to calculate the local synthetic density between the original and generated data is shown.

Fig 5 illustrates the principle of calculating local density. The generator of the GANs first fits the distribution of the original data, generating fake data *FD* with objects distributed similarly to normal objects. *N*_*k*_(*X*_*i*_) represents the neighborhood set composed of the *k*-nearest neighbors of *X*_*i*_.

**Method 2** Local synthetic density

**Input**: Original dataset ***X***, fake data ***FD***, the number of *k*-nearest neighbor *k*

**Output**: Local synthetic density **LSD**

1. **for** *t* = 1:*m* **do**

2. **for** *k* = 1:*K* **do**

3.  Sample *xi* from *X*.

4.  Sample *FDi* from *FD*.

5. $dist(X_{i},FD_{i})=\sqrt{(X_{i1}-FD_{i1}{)}^{2}+(X_{i2}-FD_{i2}{)}^{2}+,\ldots ,+(X_{id}-FD_{id}{)}^{2}}$

6. **end for**

7. #Construct the neighborhood of *Nk*(*Xi*)

9. $LSD(x_{i})=\frac{1}{\sum_{{FD}_{j}\in N_{k}(x_{i})}dist(x_{i,}{FD}_{j})}$

10. **end for**

11. **return** Local synthetic density

### 3.3 Anomaly factor

The anomaly factor(*AF*) represents the degree of deviation of an object from others; the higher the value, the more likely the object is to be an anomaly object. The method for calculating the anomaly factor is defined as shown in Eq 7.


$$AF_{i}=\frac{\sum_{FD_{i}\in N_{k}(X_{i})}\frac{LSD(FD_{i})}{LSD(X_{i})}}{\left|N_{k}(x_{i})\right|}$$ (7)

In Eq 7, *AFi* is the anomaly factor of the *i*-th object in original data *X*, *Nk*(*xi*) is the number of synthetic neighbors. If the similarity in average local density between an object in the original data and its synthetic neighbor objects is higher, it is less likely to be an anomaly, and its *AF* value will tend to be closer to 1. If the *AF* value approaches 0, it indicates that the object is located in a high-density area, reducing the likelihood of it being an anomaly. If the *AF* value is greater than 1, it indicates that the object is in a sparser area compared to the generated "normal objects," making it more likely to be an anomaly object.

## 4. Experiments

This section provides a detailed description of the experimental setup, results, and in-depth analysis. It outlines the methodologies used for evaluating the proposed methods, the datasets employed, and the experimental procedures followed to ensure robustness and reliability.

### 4.1 Experimental design

The experimental design section provides an overview of the experimental design, detailing the baseline methods used for comparison, the datasets employed, and the evaluation metrics applied. These elements are essential for systematically assessing the performance and effectiveness of the proposed method.

#### 4.1.1 Comparison methods

To verify the effectiveness of the proposed GALD method in anomaly detection tasks, five widely validated and efficient methods from the anomaly detection field were selected for comparison. These methods and their hyperparameter settings are as follows:

- Autoencoder (AE), Multi-Objective Generative Adversarial Active Learning (MO-GAAL), Single-Objective Generative Adversarial Active Learning (SO-GAAL), STEP-GAN and f-AnoGAN. The learning rates for these methods are set to 0.0001, and the hidden layers are both set to 3, ensuring that they have equivalent learning capabilities.
- Local Anomaly Factor (LOF), a method based on local anomaly factors. To ensure a fair comparison with the proposed methods, we varied the *k*-value for LOF in the range from 1 to 100 and selected the best result.
- K-means, an method based on clustering. The number of clusters (*k*) in the *k*-means method requires manual setting; in this study, we varied *k* within the range of 1 to 20 based on the dataset used.
- K-Nearest Neighbors (KNN), a distance-based method. Similar to LOF, KNN considers the number of neighbors during the detection process. We searched for the optimal *k*-value for KNN in the range of 1 to 100.
- solation Forest (IForest), an isolation-based method. In the detection process of Isolation Forest, sampling is required for the data set under investigation [37]. In this study, we set the sampling range to 10 to 200.

For proposed GALD method, we applied strict parameter settings. To ensure that GALD has comparable neural network learning capabilities with benchmark methods, the number of hidden layers is set to 3 and the learning rate set to 0.0001. For the calculating local density module and anomaly factors module, the hyperparameter values in the range of 1 to 100.

#### 4.1.2 Real-world datasets and evaluation method

Real world benchmark datasets are an effective way of comparing the detection capabilities of different anomaly detection methods. ODDS (a widely used public database in the field of anomaly detection) provides a wealth of benchmark datasets for this purpose. In this study, we use several types of benchmark datasets in ODDS to evaluate the performance of the proposed method.

Before applying the proposed method, several preprocessing steps were carried out to ensure data quality and comparability. Specifically: (1) Deduplication. The datasets were deduplicated to remove redundant entries, ensuring that only unique samples were retained. This step helps to avoid bias introduced by repeated data and improves the reliability of the evaluation. (2) Normalization: We applied Min-Max normalization to bring all features into the range [0, 1]. This technique rescales each feature according to its minimum and maximum values using the Formula 8:

$$x'=\frac{x-x_{\min}}{x_{\max}-x_{\min}}$$ (8)

where *x* is the original feature value, *x*_min_ and *x*_max_ are the minimum and maximum values of that feature. Min-Max normalization was used because it ensures all features are on the same scale, as features with larger magnitudes could otherwise dominate the results. This step helps improve the stability of the GAN training process by preventing any one feature from having a disproportionate influence.

1. **Breastw Dataset:** This dataset is derived from fine needle aspiration images of breast lumps and mainly describes the morphological characteristics of cell nuclei, with 9 dimensions and a total of 683 objects, including 239 anomaly objects. The information it contains is used to analyze the nature of breast lumps.
2. **Heart Dataset:** This dataset extracts features from regions of interest in heart images taken under different conditions, such as rest and stress. These features reflect the activity levels in different heart states. The dataset has 44 dimensions and a total of 267 objects, including 55 anomaly objects.
3. **Glass dataset:** This dataset consists of glass fragment samples collected from crime scenes, categorized by analyzing the chemical composition of the glass. It contains 9 dimensions and a total of 213 objects, including 9 anomaly objects. The data reflects the compositional differences between various types of glass.
4. **Pima Dataset:** This dataset records multiple biological characteristics related to diabetes in a Native American population from Arizona. These features can be used to study the risk of individuals developing diabetes. It contains 8-dimensional features, totaling 768 objects, with 268 anomaly objects.
5. **Inner Race, Outer Race, Ball Fault Dataset:** These dataset from the bearing fault diagnosis laboratory at Case Western Reserve University. The dataset describes 3 main types of bearing failures. It contains 23 dimensions, totaling 860 objects, with 60 anomaly objects. These datasets extract time-frequency domain features from bearing vibration signals, reflecting different wear conditions of the bearings. They provide specific signal characteristics related to bearing faults.

The evaluation method of the experimental results are crucial for determining the final performance of the method. We choose five commonly used metrics in the field of anomaly detection to evaluate the proposed method and comparison methods. These evaluation methods are (i) Area Under Curve (AUC), which is commonly used to measure the performance of classification models for binary classification tasks. A higher AUC value indicates better classification performance, especially in imbalanced datasets; (ii) Execution time. This metric refers to the time taken for the algorithm to complete its processing and return results. It is crucial for assessing the efficiency and real-time applicability of the method; (iii) Accuracy (ACC). Accuracy represents the proportion of correctly classified objects (both true positives and true negatives) among the total objects. It gives an overall measure of how well the method performs in terms of correct predictions; (iv) Detection rate (DR). Detection rate, also known as the true positive rate or recall, measures the proportion of actual anomalies that are correctly identified by the method. A higher detection rate indicates better sensitivity in anomaly detection; (v) False alarm rate (FAR). The false alarm rate represents the proportion of normal objects that are incorrectly classified as anomaly objects (false positives) out of all normal objects.

### 4.2 Experimental results and analysis

In this section, we first present the visual detection results of the GALD method. Then, we analyze the final experimental results of the proposed method and the comparison methods based on five evaluation metrics. Finally, we provide an analysis and summary of the underlying patterns revealed by the experimental results.

**Fig 6:**
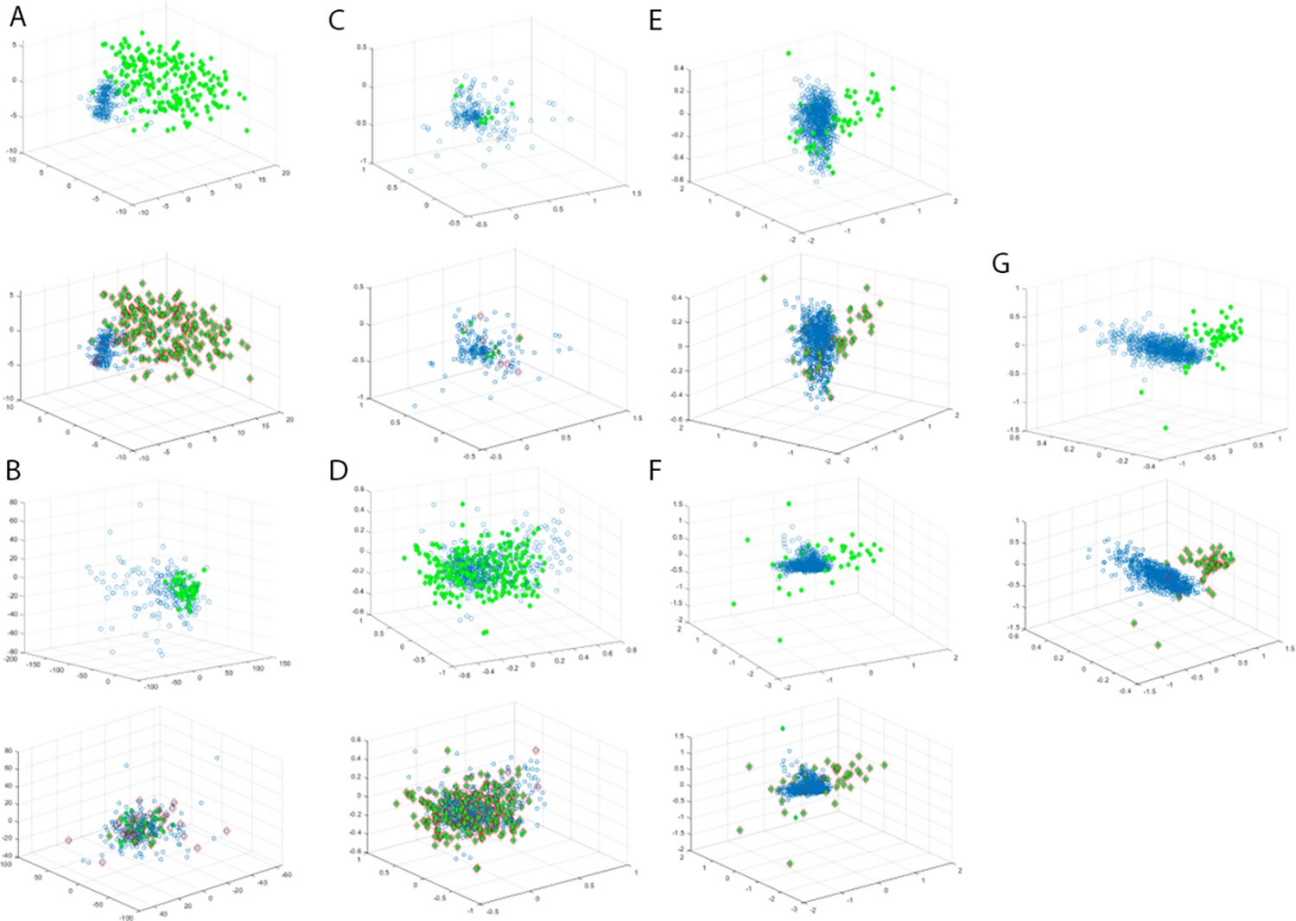
Visualization of GALD method experimental results. Each subfigure corresponds to the detection results of different datasets. The left subfigure shows the visualization results of the original dataset, and the right subfigure shows the detection results of the GALD method, which shows the anomaly detection ability of the GALD method by comparison.

Observing Fig 6, some interesting information about the GALD detection of anomaly objects are summarize as follows:

(i) Across the seven datasets mentioned above, there is no clear boundary between normal and anomaly objects. A few anomaly objects are located around or within the region of normal objects. The GALD method combines the ability to fit the data distribution with generative adversarial networks and the density of objects with their neighbors. This comprehensive approach allows it to accurately detect even mildly deviated anomaly objects.

(ii) In datasets like breastw, pima, inner race fault, outer race fault, ball fault, etc., GALD demonstrates highly competitive detection performance. This is because the distribution pattern of normal objects in these datasets is relatively easy to fit. In contrast, the distribution pattern of normal objects in other datasets appears more scattered, making it challenging for the GALD method to fit them accurately. For instance, in the heart dataset and glass dataset, the difficulty primarily arises from the inherent properties of these datasets. The heart dataset has a high dimensionality and complex feature relationships, making it hard to differentiate between normal and anomaly objects. Furthermore, in both the heart and glass datasets, the distributions of normal and anomalous objects have significant overlap, which reduces the distinguishable features available for effective anomaly detection. Similarly, the glass dataset has a small number of samples and subtle differences between classes, resulting in fewer clear patterns for the model to learn. These characteristics make anomaly detection in these datasets more challenging, contributing to the lower performance of the GALD method on these datasets.

(iii) Nevertheless, GALD exhibits considerable robustness and accuracy when dealing with datasets characterized by complex distribution patterns. By harnessing the powerful fitting capabilities of generative adversarial networks, GALD can identify a broader range of anomaly objects, even when these anomalies are embedded within intricate normal objects distribution patterns.

Through the analysis of Table 4, the following key insights can be distilled:

i): The GALD method achieved the best AUC values in six out of the seven datasets. Regarding the generalization performance of each method, GALD shows a better generalization ability. The stronger the generalization capability, the more stable and reliable the method’s performance across different datasets and tasks. This experimental result also indicates that GALD has a higher adaptability. Even in the face of different data distributions, GALD maintains a high level of detection accuracy.

ii): The GALD method does not have an advantage in terms of execution time. This is mainly because GALD detection process involves many matrix computations, includes training of the network and finding nearest neighbors, which reduces the overall efficiency of GALD. However, this problem is not intolerable. Although the GALD method has a relatively longer execution time, the improvement in detection performance justifies this trade-off. Specifically, GALD achieved an average AUC of 0.874 across 7 datasets, which is 7.2% higher compared to the next best-performing method. Additionally, GALD demonstrated an accuracy of 94.34%, an average Detection Rate (DR) of 75.16%, and a False Alarm Rate (FAR) of 3.52%. These metrics indicate that GALD outperforms existing methods in key performance indicators. In practical anomaly detection tasks where accuracy is crucial—such as in healthcare or safety monitoring—the trade-off between execution time and enhanced detection capability can be deemed acceptable, as the benefits of identifying anomalies more accurately often outweigh the need for faster processing.

iii): When the GAN fits the original data distribution, due to the overwhelming number of normal objects, the generator, under the constraint of the loss function, tends to fit the distribution of normal data. This means that the generated data primarily reflects the distribution characteristics of normal objects, while anomaly objects are often overlooked. By incorporating local synthetic density, the GALD can detect distribution differences in the local regions between the original and generated data, especially since anomaly objects often exhibit low density in these local regions, making them easier to identify. This approach effectively compensates for the limitations of GANs in high-dimensional complex data distributions, making them more sensitive to anomaly objects.

**Table 4:** Experimental results. Comparison results between the GALD method and other state-of-the-art anomaly detection methods on five metrics are shown to illustrate the advantages of GALD.

Datasets	AUC																							
	GALD	AE		LOF		K-means			IForest		KNN			MO-GAAL			SO-GAAL			f-AnoGAN			STEP-GAN	
breastw	**0.973**	0.890		0.813		0.881			0.940		0.923			**0.964**			0.632			0.833			0.964	
heart	**0.662**	**0.490**		0.242		0.365			0.265		0.199			0.315			0.203			0.278			0.388	
glass	0.671	0.569		**0.794**		0.473			0.711		**0.811**			0.693			0.554			0.605			0.716	
pima	**0.959**	0.946		0.490		0.951			0.670		**0.968**			0.758			0.669			0.713			0.820	
inner race fault	**0.971**	0.932		0.710		0.958			0.923		0.658			**0.951**			0.894			0.926			0.951	
outer race fault	**0.969**	0.911		0.946		0.059			**0.966**		0.890			0.933			0.955			0.904			0.942	
ball fault	**0.915**	**0.877**		0.735		0.698			0.874		0.673			0.827			0.863			0.833			0.845	
(a) AUC																								
Datasets	ACC (%)																							
	GALD		AE		LOF			K-means		IForest			KNN			MO-GAAL		SO-GAAL				f-AnoGAN		STEP-GAN
breastw	**97.36**		94.43		68.66			68.66		96.48			94.14			**96.48**		68.66				70.71		96.48
heart	**76.77**		61.04		61.04			**65.54**		59.55			59.55			64.04		61.04				63.29		67.04
glass	**92.48**		**92.48**		92.48			92.48		92.48			92.48			92.48		92.48				92.48		92.48
pima	**96.61**		85.15		70.05			**95.83**		72.91			77.34			72.91		70.05				70.57		73.69
inner race fault	**99.30**		97.20		93.72			97.67		97.21			93.02			**98.13**		96.51				97.20		98.13
outer race fault	**99.06**		95.34		96.28			94.65		**97.91**			95.12			95.81		97.21				95.12		96.28
ball fault	**98.83**		92.79		92.79			91.39		**97.21**			91.05			92.55		96.51				92.55		92.79
(b) Accuracy																								
Datasets	DR (%)																							
	GALD		AE		LOF		K-means			IForest			KNN			MO-GAAL			SO-GAAL			f-AnoGAN		STEP-GAN
breastw	**96.24**		92.05		55.23		55.23			94.97			91.63			**94.97**			55.23			58.15		94.97
heart	**43.63**		5.45		5.45		16.36			1.81			1.81			12.72			5.45			10.90		**20.00**
glass	**11.11**		**11.11**		11.11		11.11			11.11			11.11			11.11			11.11			11.11		11.11
pima	**95.14**		78.73		57.08		**94.02**			61.19			67.53			61.19			57.08			57.83		62.31
inner race fault	**95**		80		55		83.33			80			50			**86.67**			75			80		86.67
outer race fault	**93.33**		66.67		73.33		61.67			**85**			65			70			80			65		73.33
ball fault	**91.67**		48.33		40		38.33			**80**			35			46.67			75			46.67		48.33
(c) Detection rate																								
Datasets	FAR (%)																							
	GALD		AE		LOF		K-means			IForest		KNN			MO-GAAL			SO-GAAL			f-AnoGAN			STEP-GAN
breastw	**2.02**		4.27		24.09		24.09			2.70		4.50			**2.70**			24.09			22.52			2.70
heart	**14.62**		24.52		24.52		**21.69**			25.47		25.47			22.64			24.52			23.11			20.75
glass	**3.92**		**3.92**		3.92		3.92			3.92		3.92			3.92			3.92			3.92			3.92
pima	**2.60**		11.40		23.00		**3.20**			20.80		17.40			20.80			23.00			22.60			20.20
inner race fault	**0.37**		1.50		3.37		1.25			1.5		3.75			**1.00**			1.87			1.50			1.00
outer race fault	**0.50**		2.50		2.00		2.875			**1.12**		2.62			2.25			1.50			2.62			2.00
ball fault	**0.62**		3.87		4.50		4.62			**1.51**		4.87			4.00			1.87			4.00			3.87
(d) False alarm rate																								
Datasets	Time (second)																							
	GALD		AE		LOF		K-means			IForest		KNN			MO-GAAL			SO-GAAL			f-AnoGAN			STEP-GAN
breastw	0.177		0.155		0.153		**0.124**			0.169		**0.134**			0.214			0.168			0.177			0.238
heart	0.154		0.099		0.079		**0.020**			0.228		**0.016**			0.163			0.091			0.104			0.179
glass	0.105		0.062		0.082		**0.007**			0.128		0.058			0.076			**0.045**			0.075			0.102
pima	0.236		0.194		0.213		**0.036**			**0.172**		0.174			0.323			0.195			0.259			0.299
inner race fault	0.633		0.313		**0.057**		**0.077**			1.214		1.674			0.943			0.298			0.563			1.035
outer race fault	0.594		0.346		**0.064**		**0.059**			1.097		1.117			0.822			0.197			0.446			0.908
ball fault	0.738		0.359		**0.059**		**0.065**			0.986		1.341			1.504			0.933			1.228			0.982
(e) Execution time																								

## 5. Conclusion

In response to the difficulty of existing methods to learn the distribution patterns of the data to be detected in an unsupervised scenario, leading to low accuracy in detecting anomaly objects, we propose an anomaly detection method based on GANs and local synthetic density. The method first utilizes GANs to learn the original data distribution; then calculates the local density between the original data and the synthetic data; finally, the greater the deviation of an object’s density compared to its synthetic neighbors, the more likely it is to be an anomaly object. This method enables GALD to utilize the distribution fitting capabilities of GANs and enhances the correlation analysis among data objects, offering a valuable new perspective for anomaly detection. Extensive experiments show that GALD method is significantly superior to the comparison methods in terms of detection accuracy and generalization, proving the reliability of the GALD method. In future work, we will further optimize the diversity of the synthetic data and improve the detection efficiency of the method. Specifically, we plan to implement approximate nearest neighbor search strategies, instead of exact nearest neighbor search, which is computationally expensive, approximate methods (e.g., using KD-trees or locality-sensitive hashing) can be utilized to improve efficiency while maintaining acceptable accuracy.
